# Lens Fibrosis: Understanding the Dynamics of Cell Adhesion Signaling in Lens Epithelial-Mesenchymal Transition

**DOI:** 10.3389/fcell.2022.886053

**Published:** 2022-05-17

**Authors:** Aftab Taiyab, Judith West-Mays

**Affiliations:** Department of Pathology and Molecular Medicine, Health Sciences Centre, McMaster University, Hamilton, ON, Canada

**Keywords:** ocular lens, transforming growth factor β, epithelial to mesenchymal transition, fibrosis, cell adhesion

## Abstract

Injury to the ocular lens perturbs cell-cell and cell-capsule/basement membrane interactions leading to a myriad of interconnected signaling events. These events include cell-adhesion and growth factor-mediated signaling pathways that can ultimately result in the induction and progression of epithelial-mesenchymal transition (EMT) of lens epithelial cells and fibrosis. Since the lens is avascular, consisting of a single layer of epithelial cells on its anterior surface and encased in a matrix rich capsule, it is one of the most simple and desired systems to investigate injury-induced signaling pathways that contribute to EMT and fibrosis. In this review, we will discuss the role of key cell-adhesion and mechanotransduction related signaling pathways that regulate EMT and fibrosis in the lens.

## Introduction

The vertebrate ocular lens is a highly specialized transparent tissue of ectodermal origin that separates the anterior of the eye from the posterior. The lens is encased in its own basement membrane and is mainly composed of epithelial cells, which occupy the anterior part of the lens, and the fiber cell mass that makes up the remaining lens volume. Any perturbation in the structural organization or function of these cells results in opacification of the otherwise transparent ocular lens, causing a cataract. Cataract continues to be the second largest cause of visual impairment leading to blindness across the world, affecting nearly 94 million people with an overall financial burden of US $6.9 billion (Pascolini and Mariotti, 2012; WHO, 2012). Currently, surgical removal of the cataractous lens and its replacement by an intraocular lens (IOL) is the most common procedure performed to cure this pathological condition. Although advances in IOL design have reduced the incidence of post-surgical complications including posterior capsule opacification (PCO), delayed onset of PCO remains a significant problem (West-Mays and Sheardown, 2010; Konopinska et al., 2021; Wormstone et al., 2021). PCO involves a fibroproliferative response in which remnant lens epithelial cells (LECs) found in the capsular bag following surgery proliferate and migrate to the posterior capsule, where they undergo epithelial-mesenchymal transition (EMT) and deposit matrix (West-Mays and Sheardown, 2010; Konopinska et al., 2021; Wormstone et al., 2021). The deposition of aberrant matrix as well as cellular contraction can lead to capsular wrinkling and opacities disrupting vision. Injury to the ocular lens as occurs in primary cataract surgery results in a disruption in the cell adhesion of lens epithelial cells to each other and to their native basement membrane, the lens capsule (Konopinska et al., 2021; Wormstone et al., 2021). This disruption leads to the activation of a myriad of signaling pathways involved in normal wound healing and in lens fibrosis. This review is focused on highlighting the role of key cell-cell and cell-matrix adhesion molecule signaling pathways that contribute to the induction and progression of lens EMT and fibrosis.

## Cell Adhesion Changes and Activation of TGFβ in Lens Fibrosis

TGFβ is a cytokine that has been shown to be a key modulator of the fibrotic cataracts PCO and anterior subcapsular cataract (ASC). Indeed, the aqueous humor from patients having undergone cataract surgery exhibits increased levels of active TGFβ (Wallentin et al., 1998), and TGFβ-induced signaling has been observed in injured lens epithelial cells (LECs) (Saika et al., 2002). Numerous experimental animal models and primary cell cultures have shown that exogenous (active) TGFβ can promote lens EMT and the formation of myofibroblasts as occurs in PCO and ASC (Novotny and Pau, 1984; Lovicu et al., 2004; West-Mays and Sheardown, 2010). Canonically, TGFβ signaling functions through the activation of Small Mothers Against Decapentaplegic (Smad) proteins. Binding of TGFβ to its receptor results in phosphorylation of Smad2/Smad3, which in a complex with Smad4, translocates to the nucleus where they modulate the expression of TGFβ-responsive genes (Shi and Massague, 2003). In the lens, TGF-β induced fibrosis has been shown to occur *via* Smad-dependent pathways (Saika et al., 2002; Banh et al., 2006; Shirai et al., 2006; Meng et al., 2018). However, multiple experimental models of TGF-β-induced EMT in the lens have also revealed the contribution of non-canonical TGF-β signaling pathways including the β-catenin and Rho/ROCK mediated pathways (Maddala et al., 2003; Lovicu et al., 2015; Korol et al., 2016; Taiyab et al., 2016).

Although active TGFβ has been shown to be a major inducer of lens EMT and fibrosis, lens injury studies suggest that earlier, upstream events are required for activation of TGFβ and its sustained expression (Jiang et al., 2018). The secreted TGFβ ligand is believed to be typically stored as a latent complex with its prodomain interacting with latent TGFβ binding protein 1 (LTBP1) and latent binding peptide (LAP) in the ECM of the lens (Shihan et al., 2017; Shihan et al., 2020). During normal wound healing, as occurs in the lens post-surgery, LECs on the remaining capsule deposit a provisional matrix that includes fibronectin (FN), tenascin C, and collagen I, ECM molecules that are also involved in later fibrotic events (Rousselle et al., 2019). Of these molecules, FN has received much attention because of its role in wound healing and association with lens epithelial cell fibrosis. Earlier studies had shown that lens cells in human post-surgery capsular bags were found to be embedded in FN (Linnola et al., 2000) and cellular FN (cFN) was found to be associated with the lens capsule and in explanted IOLs 7–8 years after surgery (Saika et al., 1998). More recent work using chick lens cultures has shown that exposing these cells to plasma FN, as would occur with wounding during surgery, resulted in the activation of latent TGFβ (VanSlyke et al., 2018). Shihan and others (Shihan et al., 2020) have shed further light on the requirement for FN in lens fibrosis and how it plays an upstream role of TGFβ by creating a conditional knockout of cFN in the developing lens of mice and subjecting them to lens injury. While wild-type mice showed a fibrotic response 3 days following surgery, the FNcKO mice exhibited a significantly attenuated fibrotic response. Interestingly, reduced TGFβ upregulation was also observed in the FNcKO mice and when exogenous, active TGFβ was provided the attenuated fibrotic response was rescued. These findings, demonstrate the importance of FN and its ability to modulate TGFβ signaling in driving lens fibrosis. Since increased/activated TGFβ is known to upregulate target genes such as FN and FN-binding integrins (discussed below) as well as TGFβ itself, a chronic feedback loop is thought to exist that can further exacerbate lens fibrosis (VanSlyke et al., 2018).

The main receptors that LECs use for adhering to the lens capsule are the integrins (Duncan, 2004; Wederell and De Iongh, 2006; Walker and Menko, 2009), which are comprised of heterodimers with an *a* and *β* subunit, including 18 *a* and eight *β* subunits identified in mammals that can partner to form 24 different integrin receptors that bind specific ligand or set of ligands (Duncan, 2004; Wederell and De Iongh, 2006; Walker and Menko, 2009). Since integrins are known to act as bi-directional signaling receptors performing both “inside-out”, (transmitting signals from within the cells to the integrin activity on the cell surface) and “outside-in” (transmitting extracellular signals into the cell) (Duncan, 2004; Wederell and De Iongh, 2006; Walker and Menko, 2009) signaling, it is not surprising their disruption during injury results in activation of signaling cascades involved in wound healing and fibrosis in the lens.

Duncan and others have shown an upregulation of α5β1 and several αV integrins post-surgery in mice for up to 5 days (Shihan et al., 2020). In this case, a lens injury model was performed, which mimics cataract surgery, and fibrotic markers were assessed. The authors showed that FN conditional KO (FNcKO) mice did not exhibit an upregulation of fibrotic markers like their wild-type littermates and further demonstrated that levels of phosphorylated focal adhesion kinase (pFAK), an important signaling molecule of integrin activity, were also significantly lower in the FNcKO mouse lenses post-surgery. These findings further revealed the importance of FN and its interaction with integrins in lens fibrosis.

Active TGFβ has also been shown to regulate integrin expression during lens fibrotic events. In human lens epithelial explant cultures, α5integrin was found to be upregulated by TGFβ (Dawes et al., 2007; Dawes et al., 2008). This is not surprising given its ligand, FN, is upregulated in these models and the interaction of this integrin with FN is thought to contribute to EMT and associated αSMA expression. In addition to alpha5, alpha11, alphaV and beta5 were also found to be markedly increased in response to TGFβ (Dawes et al., 2007). Plaque cells from patients with ASC also exhibit a co-localized expression of α5β1 with FN and αSMA (Yoshino et al., 2001). In other models of lens fibrosis, the αV integrins are also upregulated following TGFβ-induced EMT (Walker and Menko, 2009). Finally, integrin linked kinase (ILK), a serine-threonine kinase that binds to the cytoplasmic tails of β1, β2, and β3 subunits, is weakly expressed in the lens but has been found to be upregulated in TGFβ transgenic lenses and correlated with LEC EMT (De Iongh et al., 2005; Weaver et al., 2007). ILK also colocalizes with α5β1 and this was enhanced in the presence of FN suggesting that ILK may be involved in EMT *via* this interaction (Weaver et al., 2007).

**FIGURE 1:**
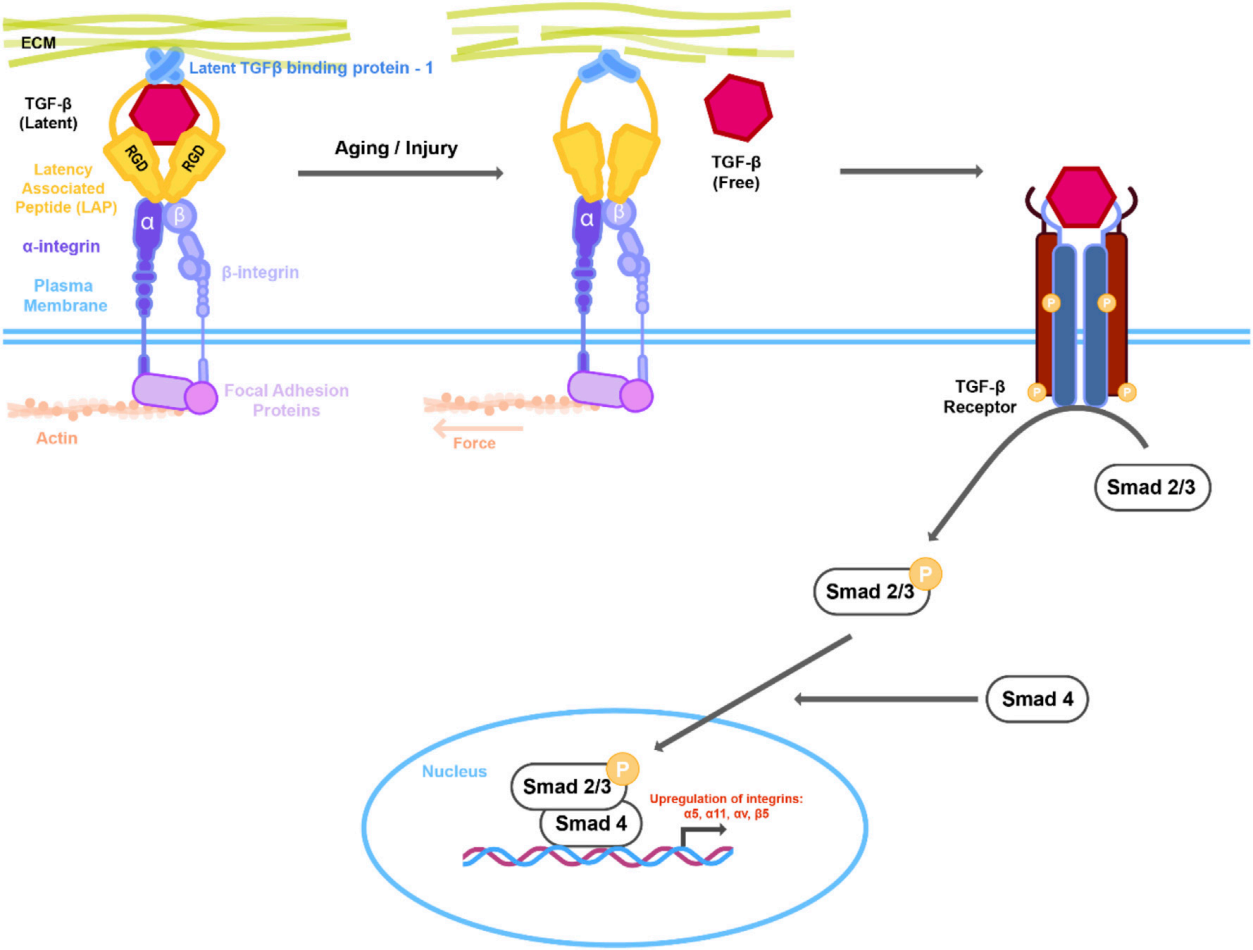
Mechanical changes in actin cytoskeleton or extracellular matrix (ECM) lead to activation of TGFβ signaling. Latent TGFβ is stored in the ECM together with the latent TGF- β1 binding protein (LTBP-1) and the latent associated peptide (LAP) that in turn is complexed with integrins. Changes in the actin/myosin mediated cell contraction or ECM architecture due to aging or injury results in putative conformational change in the LTBP-1/LAP complex leading to release of TGFβ. Interaction of active TGFβ with its receptor activates downstream Smad signaling. Active Smad2/3 in complex with Smad4 then translocates to the nucleus resulting in upregulation of downstream genes including integrins.

Injury by mechanical trauma is thought to modulate expression of the αV integrin, which has relevance to the fibrosis that occurs after cataract surgery (PCO). Studies that have directly targeted αV integrin through conditional knockout in the murine lens have demonstrated its role in EMT and lens fibrosis (Mamuya et al., 2014). For example, following lens injury on αVcKO mice, reduced lens epithelial cell proliferation and reduced or absent fibrotic markers were detected in the mutant as compared to wild-type littermates. Further data from this study suggested that αV integrins may mediate the fibrotic response by enhancing TGF-β-mediated signaling following surgery, likely through their known roles in the activation of latent TGF-β. αVβ6 is thought to activate TGFβ through its association with an RGD peptide in the latency-associated peptide (Sheppard, 2004) and in human capsular bags αVβ6 integrin expression was shown to be increased compared to cultured, intact whole lenses that have not been injured (Figure 1) (Sponer et al., 2005). However, recent work has revealed that the β8 heterodimer of αV plays a major role in regulating injury induced fibrosis in the lens (Shihan et al., 2021). Following cataract surgery, β8 integrin–conditional knockout (β8ITG-cKO) mice exhibited an attenuated fibrotic response in the lens as compared to WT mice. Interestingly, both β5 and β6 integrin null LECs underwent fibrotic changes similar to those of WT at 5 days post cataract surgery, demonstrating that the β5 and β6 heterodimers do not play the upstream role that β8 plays in lens injury fibrosis. Further transcriptomic studies using the β8ITG-cKO lens cells showed that while WT mice exhibited upregulation of target genes of TGFβ–induced signaling following 1 day of surgery, such as integrins and their ligands, the mutants did not. Additionally, canonical TGFβ signaling (as determined by pSMAD 2/3 expression) was attenuated in the β8ITG-cKO lens. Finally, the fibrotic response in the β8ITG-cKO eyes was shown to be rescued when active TGF-β1 was given at the time of surgery. Overall, this study revealed that not only is αVβ8 integrin a major regulator of lens fibrosis post-surgery, but also does so through activation of TGFβ.

Recent studies have also demonstrated that changes in the lens capsule, the matrix of the lens, can also contribute to EMT. For example, studies have shown that advanced glycation end products (AGEs) present in the lens capsule can potentiate TGFβ2-mediated EMT in human LECs and this occurs through the upregulation of both the canonical and noncanonical pathways (Raghavan and Nagaraj, 2016; Nam and Nagaraj, 2018). Interestingly, levels of AGE were found to be higher in human lens capsules from cataractous lenses and AGE levels were also found to be age-dependent (Raghavan et al., 2016). Furthermore, in the human capsular bag model of PCO, AGE content was correlated with increased levels of TGFβ-induced αSMA (Raghavan et al., 2016). Studies have also examined the role of the receptor for AGEs, RAGE, in TGFβ-induced EMT. For example, overexpression of RAGE in the human FHL124 cell line enhanced the TGFβ2-mediated EMT response in cells when cultured on AGE-modified basement membrane (Raghavan and Nagaraj, 2016). A more recent study employing RAGE knockout (KO) mice showed that LECs isolated from RAGE KO lenses did not undergo EMT in response to TGFβ2 as the wild-type cells did and this was likely due to the reduced Smad signaling observed (Nam et al., 2021). Further lensectomy experiments performed on RAGE KO mice showed that unlike wild-type littermates that exhibited elevated levels of αSMA, FN and b1 integrin in remaining capsular bag post-surgery, the RAGE KO capsules did not (Nam et al., 2021). Overall, these findings suggest that the interaction of lens matrix AGEs with RAGE plays an important role in the TGFβ2-mediated EMT of lens and fibrosis.

## Rho/ROCK Signaling in Lens Fibrosis

Apart from the Smad signaling pathway, additional non-Smad intracellular signaling pathways, such as the RhoA/Rho-kinase pathway, have been implicated in lens fibrosis. Rho are small GTPases that switch between inactive Rho-GDP and active Rho-GTP, and are critical for the regulation of actin polymerization and organization; dysregulated actin dynamics have been linked to pathological conditions such as fibrosis (Ivanov et al., 2010). A study showed a rapid increase in RhoA activity (GTP bound form) in response to TGFβ in human LECs (FHL 124), concomitantly demonstrating the presence of stress fibers, and overlapping expression of αSMA (Korol, 2012). The key downstream effectors of RhoA pathway are Ras-related C3 botulinum toxin substrate 1 (Rac1) and Rho-associated coiled-coil containing kinases (ROCK) (Bishop and Hall, 2000). ROCK facilitates the interaction of myosin with filamentous F-actin through phosphorylation of the myosin light chain (MLC) regulatory units of Myosin II, and thus plays an important role in generation of actomyosin contractile forces, enabling alterations in cellular morphology and motility (Tan et al., 1992; Turner, 2000; Vicente-Manzanares et al., 2009). Modulation of RhoA signaling through ROCK activation has been shown to be associated with TGFβ-induced EMT-mediated fibrosis in a number of *in vitro* and *in vivo* model systems (Bhowmick et al., 2001; Masumoto et al., 2001; Tian et al., 2003; Tavares et al., 2006; Zhang et al., 2013). The study by Maddala and others was the first to establish the direct correlation between increased RhoA signaling and EMT in the lens. The authors showed that Y-27632, a specific inhibitor of ROCK-mediated RhoA signaling, prevented TGFβ-induced formation of actin stress fibers and focal adhesions in the human LEC cell line SRA01/04, (Maddala et al., 2003). Furthermore, Y-27632 prevented TGFβ-induced αSMA expression, the actin isoform that contributes to generation of mechanical tension in highly contractile myofibroblasts during EMT (Cho and Yoo, 2007).

During Rho/ROCK mediated actin polymerization, the globular (G) -actin assembles to form filamentous (F) –actin. During this process, actin binding proteins (ABPs) including myocardin-related transcription factors (MRTFs) dissociate from G-actin complex and translocate to the nucleus. Within this compartment, MRTF forms a complex with serum response factor (SRF) to regulate the expression key EMT genes (Small, 2012). Therefore, the subcellular localization of MRTF is tightly regulated by the state of actin polymerization, and thus Rho/ROCK activation. The reduction in MRTF-A, the MRTF isoform responsive to TGFβ signaling, is known to reduce matrix-stiffness, αSMA expression and scarring in various models of fibrosis (Small et al., 2010; Crider et al., 2011; Luchsinger et al., 2011; Minami et al., 2012). Using rat lens epithelial explants, Gupta and others demonstrated the correlation between actin polymerization, nuclear translocation of MRTF-A, and αSMA expression during TGFβ-induced EMT (Gupta et al., 2013). A well-known actin-MRTF-A stabilizing drug, latrunculin B, prevented nuclear translocation of MRTF-A and *a*-SMA expression in TGFβ-treated rat lens epithelial explants. On the other hand, cytochalasin D, a G-actin sequestering drug, facilitated nuclear translocation of MRTF-A (Gupta et al., 2013). A follow up study by Korol et al. established the direct link between Rho/ROCK and MRTF-A signaling in LECs upon stimulation with TGFβ (Korol et al., 2016). These authors showed that the inhibition of Rho/ROCK signaling by Y-27632 prevented TGFβ-induced nuclear translocation of MRTF-A and αSMA expression while MRTF-A inhibition by CCG-203971, a specific inhibitor that blocks nuclear translocation of MRTF-A, suppressed TGFβ-induced αSMA expression and E-cadherin degradation (Korol et al., 2016). These observations are of particular interest as inhibition of either RhoA or MRTF-A signaling prevented EMT in LECs in the presence of active TGFβ thereby showing the importance of non-canonical signaling during EMT in the lens. In other ocular tissues including the trabecular meshwork (TM) cells of the anterior angle, the Rho/MRTF-A/SRF signaling cascade has also been associated with *a*-SMA expression and increased cell contractility (Clark et al., 2005; Rao et al., 2005; Pattabiraman et al., 2015).

In addition to TGFβ, injury to the lens also activates RhoA signaling in LECs. Following mock cataract surgery in mice, pMLC2 regulates the coordinated migration of LECs on the lens capsule indicating a role of Rho kinase in LEC migration (Menko et al., 2014b). The phosphorylation of MLC2 is the key factor in stress fiber formation, and actomyosin contractility. Rho kinase phosphorylates MLC2 directly thereby stimulating the cross-linking of actin by myosin leading to enhanced cell contractility (Katoh et al., 2011). Tanaka *et al* performed a series of immunohistochemistry (IHC) analysis on lenses from mice upon needle injury. The lens epithelium of these mice showed increased expression of TGFβ1, fibronectin and *a*-SMA. As expected, the epithelium of injured lenses also showed increased activation of MLC9, and thus Rho signaling, suggesting an important role of Rho kinase signaling in the induction of EMT in lens upon injury (Tanaka et al., 2010). In continuation, the group performed a detailed study using a similar model system and showed that systemic administration of fasudil hydrochloride, a specific inhibitor of Rho kinase signaling, prevented LEC proliferation, capsule contraction, and MRTF-A nuclear translocation (Ichikawa et al., 2020). To corroborate their observations, they performed similar assays upon systemic administration of CCG-203971 in mice with injured lenses. The lens epithelium of mice showed decreased contraction of the capsule and suppressed MRTF-A nuclear translocation upon systemic administration of CCG-203971 (Ichikawa et al., 2020).

**FIGURE 2:**
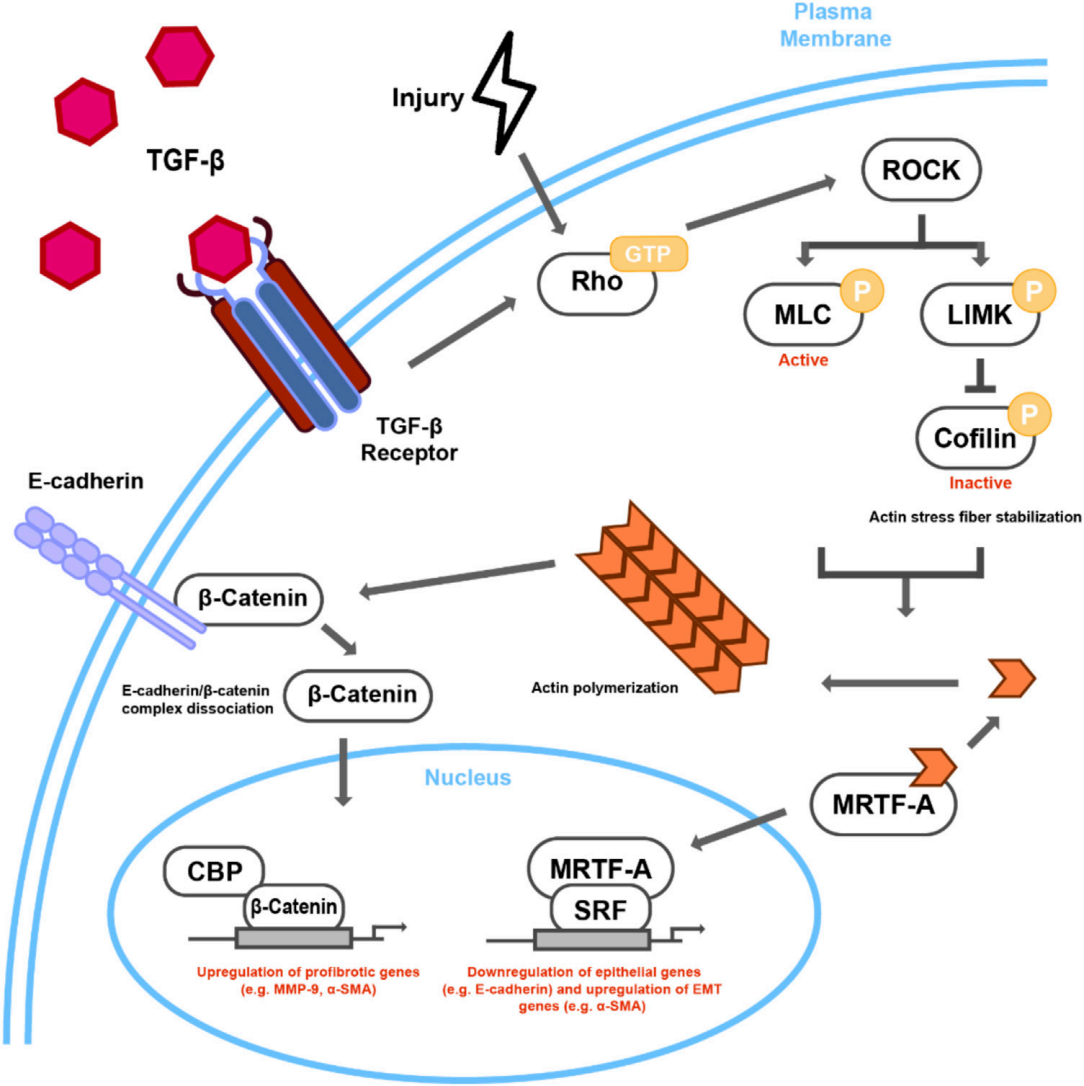
Proposed mechanism of Rho-dependent cytoskeletal signaling in TGFβ/Injury-induced lens EMT. TGFβ stimulation or injury leads to Rho-GTP regulated ROCK activation. ROCK leads to actin stress fiber formation and actomyosin contractility through phosphorylation of both MLC and LIMK, the latter of which phosphorylates cofilin, rendering it inactive. Incorporation of G-actin monomers into contractile stress fibers leads to the nuclear accumulation of MRTF-A, which when in complex with SRF, can activate the transcription of EMT-related targets, such as αSMA. The interaction of stress fibers with E-cadherin can then destabilize E-cadherin/β-catenin complex leading to nuclear transcriptional activity of β-catenin, specifically through CBP and other unknown transcription factors.

One of the important outcomes of Rho kinase signaling is actin cytoskeletal remodeling, which mainly occurs through polymerization of G-actin into F–actin fibers. F-actin, along with actomyosin, forms stress fibers that upon its interaction with focal adhesion points and cell junctions play an important role in cell motility, shape, and morphogenesis (Anderson et al., 2008). Epithelial cells are held together by three major types of junctional complexes: tight junctions (TJs), adherens junctions (AJs), and desmosomes; they are also connected to the ECM through integrins (Yilmaz and Christofori, 2009). E-cadherin, the major component of AJ, is attached to the actin cytoskeleton and mediates cell-cell adhesion complexes *via* β-catenin and *a*-catenin (Noren et al., 2000; Noren et al., 2001). The formation of contractile stress fibers destabilizes the E-cadherin, β-catenin and *a*-catenin complex at the AJs, leading to internalization and degradation of E-cadherin as well as activation, and subsequent nuclear translocation, of β-catenin in the LECs during EMT (Korol et al., 2016; Taiyab et al., 2016; Taiyab et al., 2019). Stabilization of Rho signaling *via* inhibition of ROCK prevented formation of stress fibers and the delocalization and degradation of E-cadherin, thereby preventing nuclear translocation of β-catenin and abrogating EMT in LECs (Figure 2) (Korol et al., 2016). In addition to AJs, Rho-kinase induced stress fibers also form a key component of the multi-protein integrin-mediated cell-matrix adhesion complex that is achieved through interaction of integrins with actin cytoskeleton *via* cytoskeletal linker proteins such as talin, paxillin and vinculin (Burridge and Guilluy, 2016). The modulation of cytoskeleton dynamics during EMT results in activation of integrin-mediated signaling leading to expression of downstream EMT genes (*discussed in previous section*). Taken together, these studies show that Rho/ROCK signaling is central to EMT induction in the lens either upon injury or induced by growth factor, and therefore modulating the expression and/or activation of RhoA/ROCK signaling might serve as a possible prognosis for EMT-mediated fibrosis in the lens and PCO.

## Mechanotransduction and Lens Epithelial-Mesenchymal Transition and Fibrosis

The mechanical cues arising from changes in cell matrix adhesion complex, cellular tension, and ECM stiffness trigger activation of key mechanotransduction signaling pathways that are believed to be critical for the induction of EMT and fibrosis (Dupont et al., 2011). The core components of evolutionarily conserved Hippo pathway, Yes-associated protein (YAP) and its paralog, the transcriptional coactivator with PDZ-binding motif (TAZ), are believed to be central to mechanotransduction signaling pathways (Piccolo et al., 2014). Epithelial cells when stretched by a stiff ECM showed increased cell spreading, as well as nuclear localization and elevated transcriptional activity of YAP/TAZ, facilitated by actomyosin contraction, focal adhesions, and stress fiber formation (Dupont et al., 2011). Under these conditions, the nuclear translocation of YAP/TAZ is solely dependent on mechanical cues and is not influenced by Hippo signaling pathway (Dupont et al., 2011). Using whole lens culture, Kumar et al showed nuclear translocation of YAP and increased LEC proliferation in lenses upon administration of mechanical stress that was prevented by verteporfin, an inhibitor that blocks nuclear translocation of YAP, thereby showing a direct correlation between LEC proliferation and YAP signaling (Kumar et al., 2019). A recent study showed that knockdown of acidic calponin (CNN3), a well-characterized actin, myosin, tropomyosin, and calcium/calmodulin binding contractile protein, resulted in reorganization of actin stress fibers, increased focal adhesions, and enhanced YAP/TAZ transcriptional activity, leading to mouse LEC transdifferentiation (Maddala et al., 2020).

The crosstalk between YAP/TAZ, focal adhesion, and Rho kinase signaling is also important for the induction of EMT. YAP/TAZ plays an important role in focal adhesion signaling by modulating interaction of focal adhesions (FAs), mainly integrins, to the F-actin in the actin cytoskeleton and fibronectin expressed in the ECM by myofibroblasts (Winograd-Katz et al., 2014; Zent and Guo, 2018). The focal adhesions mediate force transmission between the ECM and actin cytoskeleton *via* the Rho/ROCK pathway, a pathway that is critical for EMT-induction in the lens, to inhibit phosphorylation of YAP thus facilitating its nuclear translocation (Nobes and Hall, 1995; Huveneers and Danen, 2009; Kim and Gumbiner, 2015; Burridge and Guilluy, 2016; Korol et al., 2016; Nardone et al., 2017). Recent work from our laboratory has shown that increased expression of YAP1 in lens sections from a mouse model of ASC provides further evidence of involvement of YAP/TAZ in lens fibrosis (Taiyab and coworkers, unpublished observation). Furthermore, we have found that inhibition of nuclear translocation of YAP1 by verteporfin prevented TGFβ-induced αSMA expression as well as E-cadherin delocalization and degradation in rat lens epithelial cell explants suggesting a critical role of YAP1 in lens EMT (Taiyab and coworkers, unpublished observations).

One of the major causes of EMT-mediated age-related fibrotic cataract is modulation of interaction of LECs with its basement membrane, the lens capsule, resulting from the changes in the matrix architecture of the lens capsule due to aging (Danysh et al., 2008; Danysh and Duncan, 2009). Such changes can alter the organization and rheology of the lens capsule. In addition to YAP/TAZ, Piezo1, a mechanosensitive cationic channel that opens upon physical deformations of the lipid bilayer such as increased membrane tension, has also been implicated in modulation of lens transparency (Botello-Smith et al., 2019; Allen et al., 2020). In normal cells including epithelial, fibroblast, and endothelial cells, Piezo1 is enriched at focal adhesions in a force dependent manner. Piezo1, through Calpain-dependent pathways, contributes to focal adhesion formation, turnover, and force generation, and also acts as a major sensor of mechanical cues in mechanosensing processes (Yao et al., 2020). The mouse LECs express Piezo1, which regulates calpain-mediated calcium-dependent MLC phosphorylation. Dysregulation of Piezo1 led to degradation of lens membrane protein and loss of lens transparency (Allen et al., 2020). The activity of Piezo1 is regulated by both membrane tension and membrane-associated adhesion complexes that include cytoskeletal connections. The cells with blocked Piezo1 channels show an inability to spread, a low cell volume aspect ratio, and a thin tail-like extension (Jetta et al., 2021). In the tips of the spreading cells, Piezo1 co-localizes with Paxillin, a major component of focal adhesion complexes that involves myosin-II contractility *via* Rho/ROCK pathway. Inhibition of Rho/ROCK pathway also prevented cell elongation along with a decrease in Piezo1 density at the cell extension points (Jetta et al., 2021). These studies suggest a potential, overlapping role of both YAP/TAZ and Piezo1 in modulation of cell-adhesion based signaling during lens EMT.

## Cytoskeletal Protein Mediated Signaling During Lens Epithelial-Mesenchymal Transition

Injury-induced repair results from collective migration of epithelial cells to the wounded area, a process that is controlled by partially transformed mesenchymal-like leader cells (Friedl and Gilmour, 2009). These mesenchymal-like leader cells possess projections such as lamellipodia, mainly composed of vimentin, a type III intermediate filament, which guides the movement of epithelial cells as one collective sheet/cluster to the wounded area (Friedl and Gilmour, 2009; Khalil and Friedl, 2010). Vimentin-deficient adult animals showed delayed migration of fibroblasts into the wound site and subsequently retarded contraction that correlated with a delayed appearance of myofibroblasts at the wound site (Eckes et al., 2000).

Using a mock cataract surgery model in chick, Walker et al observed increased expression of vimentin intermediate filaments in the lamellipodia of the mesenchymal-like leader or repair cell population located in the lens equatorial region (Walker et al., 2010). These cells originate from a subpopulation of cells within the lens epithelium that act as progenitors for mesenchymal repair cells through EMT. Vimentin filaments are linked, in a complex, with paxillin-rich focal adhesion and motor protein myosin IIB (Sanghvi-Shah and Weber, 2017). A reduction in vimentin expression or disruption of vimentin function by inhibitors such as Withaferin A disrupts the ability of repair cells to form lamellipodia processes at the wound edge and impairs wound closure, suggesting a critical role of vimentin in cell adhesion signaling that contributes to cell migration and proliferation (Walker et al., 2010; Menko et al., 2014a). In addition, the non-filamentous soluble form of vimentin, known to act as a long-distance messenger during wound healing, can also be seen scattered throughout the lens epithelial sheet in the mock cataract surgery model system (Menko et al., 2014a). Increased levels of soluble vimentin regulated through posttranslational modifications are associated with signaling and are correlated with the disease progression (Perlson et al., 2005; Lahat et al., 2010). While phosphorylation contributes to disassembly of intermediate filaments, citrullination (deamination) of vimentin promotes its disassembly, increasing the soluble extracellular vimentin (Inagaki et al., 1989; Teshigawara et al., 2013). A negative correlation has been established between the organized vimentin intermediate filament cytoskeletal network and Rho kinase activity. It has been shown that vimentin intermediate filaments can inhibit Rho kinase activity and block both actin stress fiber formation and myosin contractility (Jiu et al., 2017). In contrast, the soluble form of vimentin is known to contribute to the contractile phenotype of myofibroblasts.

Repair cells following mock cataract surgery show increased presence of soluble vimentin, specifically in the wound activated mesenchymal cells that localize to the leading edge of the wound. The soluble vimentin is released extracellularly in response to injury where it is known to mediate the differentiation of leader cells to myofibroblasts (Walker et al., 2018). The presence of vimentin in the punctate structures of invading wound-activated leader lens cells when plated on matrigel suggests contribution of extracellular vimentin in matrix remodeling. This might be achieved through the interaction of extracellular vimentin with cell surface receptors such as CD44 and IGF-1R, for which vimentin acts as a ligand. CD44 is a cell-surface glycoprotein involved in cell-cell interactions, cell adhesion, and migration that becomes active after being cleaved by membrane type matrix metalloproteinase (MT1-MMP). Both the extracellular and intracellular domain of CD44 has been correlated with disease progression (Senbanjo and Chellaiah, 2017). In addition to expressing extracellular vimentin, the wound-activated leader LECs also showed the increased presence of CD44 at the cell borders (Walker et al., 2018). Therefore, one possible mechanism through which extracellular vimentin might be modulating the function of CD44 is by facilitating its cleavage during wound healing in the lens.

Another mechanism through which extracellular vimentin might be contributing to the increased cell migration and invasion is FAK signaling. In the mock cataract injury model, vimentin colocalized with prominent paxillin-rich adhesion plaques at the tips of the lamellipodia of migratory leader LECs (Menko et al., 2014a). The colocalization of extracellular vimentin with activated focal adhesion kinase (FAK) in injured/stressed epithelial cells of rat lens explants suggest the role of vimentin in focal adhesion signaling (Taiyab and coworkers). Cellular mechanical stress mediated integrin clustering results in autoactivation of FAK (Parsons, 2003; Lee and Nelson, 2012). Activated FAK subsequently phosphorylates Src kinases, which in turn phosphorylates other tyrosine sites on FAK to initiate downstream signaling including the Rho/ROCK pathway that results in increased actin polymerization, cell contractility, and migration (Mitra et al., 2005). Further molecular investigations are required to reveal the mechanism(s) through which extracellular vimentin might be contributing to increased leader cell migration and invasion during injury in the lens.

## Conclusion

In recent years, there has been increased focus on understanding how injury contributes to EMT-mediated fibrosis in the lens. These studies point towards cell adhesion molecules as key players, responsible for the induction and progression of EMT in the lens, either independently or through activation of TGFβ signaling. For example, adhesion molecules such as the integrins have been shown to play an upstream role in lens induced-EMT and do so, at least in part, through activation of the conventional TGFβ signaling pathways following injury. Interaction of lens matrix AGEs with RAGE is also known to play a critical role during TGFβ-induced EMT in the lens. Furthermore, injury to the lens modulates mechanotransduction-mediated signaling including activation of YAP and Piezo1, both of which require Rho/ROCK-induced FAK signaling. FAK-mediated Rho/ROCK signaling may play an important role in enhanced cell migration, mediated by increased expression of extracellular vimentin during wound healing in the lens. As outlined in this review, many of the cell-adhesion mediated signaling pathways coordinate with one another to induce EMT and fibrosis in the lens. However, further studies are needed to understand how these complex signaling pathways crosstalk with one another during both early events of lens injury as well as in the later progression of EMT and fibrosis.
